# Genetic Association of Beta-Lactams-Induced Hypersensitivity Reactions: A Protocol for Systematic Review and Meta-Analysis

**DOI:** 10.3390/genes13040681

**Published:** 2022-04-13

**Authors:** Lalita Lumkul, Mati Chuamanochan, Surapon Nochaiwong, Mongkhon Sompornrattanaphan, Prapasri Kulalert, Mongkol Lao-araya, Pakpoom Wongyikul, Phichayut Phinyo

**Affiliations:** 1 Center for Clinical Epidemiology and Clinical Statistics, Faculty of Medicine, Chiang Mai University, Chiang Mai 50200, Thailand; lalita.lumkul@gmail.com (L.L.); zpakpoom_kc@hotmail.com (P.W.); 2 Center of Multidisciplinary Technology for Advanced Medicine (CMUTEAM), Faculty of Medicine, Chiang Mai University, Chiang Mai 50200, Thailand; 3 Division of Dermatology, Department of Internal Medicine, Faculty of Medicine, Chiang Mai University, Chiang Mai 50200, Thailand; mati.c@cmu.ac.th; 4 Pharmacoepidemiology and Statistics Research Center (PESRC), Chiang Mai University, Chiang Mai 50200, Thailand; surapon.nochaiwong@gmail.com; 5 Department of Pharmaceutical Care, Faculty of Pharmacy, Chiang Mai University, Chiang Mai 50200, Thailand; 6 Division of Allergy and Clinical Immunology, Department of Medicine, Faculty of Medicine Siriraj Hospital, Mahidol University, Krung Thep Maha Nakhon 10700, Thailand; bankallergymed@gmail.com; 7 Department of Clinical Epidemiology, Faculty of Medicine, Thammasat University, Pathum Thani 12120, Thailand; prapasrikulalert@gmail.com; 8 Division of Allergy and Immunology, Department of Pediatrics, Faculty of Medicine, Thammasat University, Pathum Thani 12120, Thailand; 9 Department of Pediatrics, Faculty of Medicine, Chiang Mai University, Chiang Mai 50200, Thailand; laoaraya@gmail.com; 10 Department of Family Medicine, Faculty of Medicine, Chiang Mai University, Chiang Mai 50200, Thailand; 11 Musculoskeletal Science and Translational Research (MSTR), Chiang Mai University, Chiang Mai 50200, Thailand

**Keywords:** beta-lactams hypersensitivity, genetic risk factor, genetic association study, polymorphism, systematic review

## Abstract

Beta-lactam (BL) antibiotics are among the drugs commonly related to hypersensitivity reactions. Several candidate gene studies and genome-wide association studies have reported associations of genetic variants and hypersensitivity reactions induced by BL antibiotics. However, the results were inconclusive. This protocol details a comprehensive systematic review of genetic factors associated with BL-induced hypersensitivity. A systematic search of literature related to genetic associations of BL-induced hypersensitivity will be performed through PubMed, Medline, Scopus, EMBASE, Web of Science, CINAHL, and the Cochrane central register of Controlled Trials (CENTRAL) from their inception dates with no language restrictions. Two reviewers will independently screen, extract, and appraise the risk of bias. Frequencies of genetic variants that comply with Hardy–Weinberg equilibrium will be extracted and pooled. Genetic models will be applied to variant effect calculation as per allele and genotype analysis. Based on statistical heterogeneity among studies, common effect estimation (odds ratio) and its corresponding 95% confidence interval will be analyzed. Sensitivity and subgroup analyses will be performed to determine the robustness of eligible studies. This systematic review and meta-analysis will provide comprehensive evidence of genetic effects regarding BL-induced hypersensitivity. The findings will enlighten the determination of disease-related genotypes that would potentially reveal allergy profiling in patients.

## 1. Introduction

Beta-lactam (BL) antibiotics are the most common drugs used for bacterial infections [1]; however, allergic reaction against BL is not uncommon. The prevalence of BL-induced allergy has been reported from 7.92% to 14.5% [2,3] in which penicillin and cephalosporin were the most common triggers [3,4]. BL-induced hypersensitivity reactions are mainly classified into immediate and non-immediate types according to the time onset after drug exposure [5]. The immediate reaction is involved in an antibody-mediated pathway that activates IgE secretion, resulting in rapid response within a few hours after exposure. The major clinical manifestations are urticaria, angioedema, and anaphylaxis [6,7]. Contrarily, non-immediate type is triggered through T-cell mediated response. The time onset is varied from more than six hours to four weeks after medication, with diverse clinical manifestations including maculopapular exanthems and severe cutaneous adverse reactions (SCARs) [8]. Despite their low incidence, SCARs, in particular, Steven–Johnson syndrome (SJS), toxic epidermal necrolysis (TEN), and drug reaction with eosinophilia and systemic symptoms (DRESS) syndrome can be life-threatening and associated with adverse sequelae and healthcare utilization [9,10,11]. With respect to the culprit antibiotics, beta-lactams were the most common drug classes that contributed to SCARs, with a range of 12.4–44.4% [12,13,14,15].

Risk evaluation of BL allergy before an antibiotic prescription is required to prevent adverse effects and extra cost of alternative treatment. Data from electronic health records of the United States revealed that 12.8% and 1.7% of the population labeled themselves as penicillin and cephalosporin allergic, respectively [3]. However, more than 95% of those were not truly sensitive to BL antibiotics and had negative skin test results [16]. Patients with a BL allergy will be treated with alternative antibiotics such as fluoroquinolone and clindamycin [17]. The use of these drugs may result in increased exposure to antibiotic-resistant pathogens and cause an economic burden due to the extension of hospital stay [4,17]. Hence, several strategies to evaluate BL hypersensitivity risk have been proposed. The tools were developed based on allergy history and a patient-assessed questionnaire which can potentially identify low-risk phenotype and de-labeling of penicillin allergy [5,18].

Accordingly, a genetic variant test has been developed to determine the risk of drug-induced hypersensitivity reactions for various medicines such as allopurinol and carbamazepine [19,20]. The affected alleles that contributed to SCARs were successfully discovered, and it is recommended to have a genetic test before a prescription to avoid severe response in patients who carry the affected genotype [21]. For BL allergy, several candidate variants have been reported, either in candidate gene studies or genome-wide association studies. Such that, several variants from interleukin 4 receptor alpha (IL4-Ra), interferon-gamma (IFN-g), human leukocyte antigen (HLA) were frequently found [22,23,24,25]. Nonetheless, the results are inconclusive. Of these, we aimed to conduct a comprehensive literature search of genetic variants and phenotypic outcomes of BL allergy to better inform the causal variants and evaluate the frequency of related clinical outcomes.

## 2. Materials and Methods

### 2.1. Study Registration

This protocol of systematic review is reported in compliance with the Preferred Reporting Items for Systematic Review and Meta-Analysis Protocols (PRISMA-P) statement [26]. The PRISMA-P checklist is provided in Supplementary Table S1. We follow guidelines proposed by the Human Genome Epidemiology Network for the systematic review of genetic association studies and PRISMA guidance [27]. The study protocol was registered in the International Prospective Register of Systematic Reviews (PROSPERO) and is currently available online (Registration number: CRD42022300283).

### 2.2. Systematic Searching of the Literature

A systematic search of relevant literature will be performed based on comprehensive search strategy guidelines [28]. The search will be conducted in collaboration with an experienced medical librarian through electronic biomedical databases and validated following the Peer Review of Electronic Search Strategies (PRESS) guideline statement [29]. The systematic search will be performed in standard biomedical databases, including PubMed, Medline, Scopus, EMBASE, Web of Science, CINAHL, and the Cochrane central register of Controlled Trials (CENTRAL) from inception with no language restrictions. The combinations of Medical Subject Headings and search terms will be used, including pharmacological class (i.e., “beta-lactam” or “penicillin” or “cephalosporin”) and genetic variants (i.e., “associations, genotype phenotype” or “allele” or “association study, genome wide” or “single nucleotide polymorphism” or “pharmacogenomics”) and the clinical phenotypes of hypersensitive reactions (i.e., “drug allergy” or “anaphylactic reaction” or “skin rash” or “hypersensitivity” or “severe cutaneous adverse reaction”). The pre-specified search strategy for each database is provided in Appendix A (Table A1, Table A2, Table A3, Table A4, Table A5, Table A6 and Table A7). Grey literature defined as unpublished records not confined to commercial publishers will be searched using Google Scholar advanced search following recommendations by Haddaway et al. to focus on title level searches [30] and other unpublished/preprint report databases (medRxiv, bioRxiv). In addition, articles from prior systematic reviews on the topic, reference lists of the included studies, ongoing clinical trials registries, and major international allergy/immunology and dermatology scientific meetings (i.e., American Academy of Allergy Asthma & Immunology (AAAAI), European Academy of Allergy and Clinical Immunology (EAACI), European Society for Dermatological Research (ESDR), Society for Investigative Dermatology (SID), British Association of Dermatologists (BAD), and American Academy of Dermatology (AAD)) will be manually browsed for further potentially relevant studies. An updated search will be conducted before the final analyses and formal dissemination by using the same set of keywords and search terms as determined in the pre-specified search strategies.

### 2.3. Selection Process and Eligible Criteria

Studies identified from the literature search will be combined and deduplicated using citation manager software. Initially, titles and abstracts will be screened using Rayyan, a web application for systematic reviews [31]; then, full-text articles will be retrieved for evaluation of eligibility by two independent reviewers (L.L and P.W.) Disagreement during the selection process will be resolved by consulting clinical experts in allergy/immunology (P.K., M.L., M.C., and M.S.) and methodologists (P.P. and S.N.). Studies will be eligible to be included if they meet the following criteria: (i) randomized trials and observational studies including case–control, cross-sectional, or cohort studies; (ii) investigated the associations of genetic markers and the risk of drug-induced hypersensitivity reactions among individuals receiving beta-lactams therapy regardless of age and indications; (iii) sufficient information provided to calculate the association between genetic variants and risk estimate (e.g., odds ratio (OR), allele frequencies). Either candidate gene studies or Genome-wide association studies (GWASs) will be included. We will exclude studies that (i) were case series/case reports, reviews, comments; (ii) had a small sample size (less than 100 participants); and (iii) investigated the association of genetic markers and other forms of drug-induced hypersensitivity phenotypes (acute interstitial nephritis, drug-induced liver injury, serum sickness, and isolated drug fever). All exclusions will be reported according to the PRISMA flow diagram.

### 2.4. Outcomes of Interest

The primary outcome was the relevant drug-induced hypersensitivity phenotypes caused by any drug classes of beta-lactams (i.e., penicillin, cephalosporin, carbapenems). The phenotypic outcome will be classified as non-immune-mediated adverse drug reaction, immune-mediated reaction, or unknown. The symptoms can vary from mild reactions, such as rash, urticaria, angioedema to anaphylaxis and severe cutaneous adverse reactions (SCARs) to drugs, including acute generalized exanthematous pustulosis (AGEP), drug rash with eosinophilia and systemic symptoms (DRESS), Stevens–Johnson syndrome (SJS), and toxic epidermal necrolysis (TEN). Ultimately, we prefer to use outcomes ascertainment based on immunological assessment (skin prick test and intradermal and/or oral provocation). For reasons of clinical relevance, however, comprehensive assessment by an allergist or dermatologist, medical record based on administrative databases, or patient’s history using a definition/questionnaire according to accepted criteria were also considered [32,33,34,35].

### 2.5. Data Extraction

Two authors (L.L. and P.P.) will independently extract data using a standardized approach and electronic extraction form. The following information will be extracted based on:

1. Study characteristics, including study authors and year of publication, geographic region and country, study design (randomized trials or non-randomized studies (cohort, case–control, or cross-sectional studies), sample size, study population (specific exposure to penicillin, cephalosporin, carbapenem, or unspecified beta-lactams), study setting and period, and statistical analysis methods.
2. Participant characteristics, including mean or median age of study population, the proportion of females, race/ethnicity, comorbid conditions, immunosuppression history, number of cases and control, and allergic risk history in both discovery and replication groups. If the ethnicity of the study population was not reported, the study location will be then used to identify the ethnicity of the particular population.
3. Genotyping-related information, including genotyping techniques (e.g., restriction fragment length polymorphism, random amplified polymorphic DNA, amplified fragment length polymorphism, polymerase chain reaction (PCR), allele-specific or sequence specific oligonucleotide probed-PCR, DNA sequencing, DNA microarrays) categories of genetic variations (i.e., single nucleotide changes, tandem repeats, short indels, or structural variations), type of genetic variation study (genome-wide association studies, studies on the functional consequence of variants, or population genetics)
4. Predefined clinical drug-induced hypersensitivity phenotypes (non-immune-mediated adverse drug reaction, immune-mediated reaction, or unknown) or specified clinical manifestation of phenotypes (e.g., anaphylaxis, angioedema/bronchospasm, SCARs, AGEP, DRESS, SJS-TEN, or maculopapular exanthema), case ascertainment definition and methods. The reported associated genetic variants and their corresponding loci will be extracted using the most current name and variant accession number according to National Center for Biotechnology Information (NCBI) databases. Affected and non-affected allele frequencies and their effect size (OR and 95% confidence interval) will be retrieved. If applicable, laboratory functional investigations (e.g., sensitivity test, drug-specific IgE response) will be provided.

For studies that have the same data source and examine the same genetic variants, only the results from the largest and the most recent will be included. Data extraction will be cross-checked by two methodologists (S.N. and P.P.). Any discrepancies during the process will be resolved by a discussion with clinical experts in allergy/immunology and dermatologist (P.K., M.L., M.C., and M.S.). For a study with missing data on the outcomes of interest, corresponding authors will be contacted via e-mail. If there is no response, the data will be reported as missing or imputed, as appropriate.

### 2.6. Quality Assessment

The quality of included studies will be assessed by two reviewers (L.L. and P.P.) independently using the recommendations framework of STrengthening the Reporting of Genetic Association Studies (STREGA) [36]. The STREGA evaluates strengths and weaknesses of evidence concerning important issues in genetic data validity and reporting of results. The total STREGA score of each included study will be reported. Any discrepancies will be discussed and consensus with the clinical experts (P.K., M.L., M.C., and M.S.) and clinical methodologists (P.P. and S.N.).

### 2.7. Data Synthesis

In the case where a meta-analysis cannot be performed owing to a limited number of included studies regarding the effect estimate of the same candidate gene, a narrative synthesis of the available evidence will be employed. If possible, the frequency of affected and non-affected variants will be extracted and pooled. A minimum of three data sources will be required for meta-analysis. The common effect estimate (odds ratio, OR) along with 95% confidence interval (95% CI) from each reported genetic variant will be calculated separately. Data will be analyzed in compliance with a type of study level (i.e., candidate gene study or GWASs).

Statistical analyses will be performed based on a genetic model for genetic association study [37]. Hardy–Weinberg equilibrium (HWE) will be primarily checked for the availability of genotyping data using a chi-square test or exact test goodness-of-fit where appropriate. Genetic variants from the studies that do not comply with HWE will not be included in the quantitative analysis. Statistical heterogeneity across studies will be evaluated using Cochrane Q-statistic and I^2^ tests. The genetic model including per allele and per genotype approach will be subsequently applied to each variant effect calculation where appropriate [38]. The genetic effect will be analyzed using a random-effects model by DerSimonian Laird method if heterogeneity is presented (Q test (*p* < 0.10) or I^2^ > 25%); otherwise, a fixed-effects model will be used. Sensitivity analyses will be assessed to determine the robustness of studies regarding their compliance with HWE. The funnel plot and Egger’s test will be tested to determine publication bias.

Subgroup analysis will be constructed based on hypersensitivity types, ethnicity, and drug classes. Statistical analysis will be performed using Stata software version 17.0 (StataCorp, College Station, TX, USA) for allele frequency analysis and effect estimation. A *p*-value < 0.05 will be considered statistically significant.

### 2.8. Assessment of Cumulative Evidence

For the meta-analysis, the Venice criteria [39] will be applied to assess the epidemiological credibility. Data will be graded based on three criteria including the amount of evidence, extent of replication, and protection from bias. The amount of evidence will be defined according to sample size, and the allele frequency of each variant. The replication will be graded by the between-study inconsistency concerning heterogeneity statistics (I^2^). Protection from bias will be considered based on the risk of bias that is likely to explain the presence of association (i.e., from genotype measurement, population stratification, selective reporting). The credibility will be determined from composite grades of three criteria and assigned as strong, moderate, or weak. Therefore, the cumulative evidence of meta-analysis will be defined as strong if all criteria are classified as A, moderate if all grades are A or B, and weak if any C grades are present.

## 3. Ethics and Dissemination

This systematic review will include data on published literature for data extraction and synthesis which does not directly involve human subjects. The ethical approval was exempted by the Ethical Committee of the Faculty of Medicine, Chiang Mai University (EXEMPTION 8794/2022, FAC-MED-2565-08794). Results from this systematic review will be reported in compliance with the Preferred Reporting Items for Systematic reviews and Meta-Analyses (PRISMA) 2020 statement guidelines [40]. Our findings will be published in peer-reviewed journals and any further amendments to the protocol will be included in the final report.

## Data Availability

Not applicable.

## Supplementary Materials

The following supporting information can be downloaded at: https://www.mdpi.com/article/10.3390/genes13040681/s1, Table S1: PRISMA-P checklist [41].

## Appendix A

**Table A1:** Pre-specified search strategy for Ovid MEDLINE(R).

Search	Query	Items Found
#1	exp penicillin/	82,792
#2	exp beta-lactam/	133,931
#3	exp cephalosporin/	44,603
#4	(penicillin* or beta-lactam* or cephalosporin*).mp.	150,865
#5	(penicillin* or aminopenicillin* or thiazolidine* or beta-lactam* or betalactam* or lactam* or cephalosporin* or aminocephalosporin* or carbapenem* or aztreonam* or monobactam*).ti,ab,kw,rn.	183,877
#6	or/1–5	225,834
#7	exp gene/	808,632
#8	exp genotype/	441,156
#9	exp phenotype/	326,826
#10	exp allele/	115,876
#11	exp genome/	1,045,997
#12	exp genome-wide association study/	35,712
#13	exp single nucleotide polymorphism/	129,480
#14	exp human leukocyte antigen/	76,067
#15	exp pharmacogenetics/	12,813
#16	(genome-wide association study or GWAS or single nucleotide polymorphism* or SNP or human leukocyte antigen or HLA or haplotype or pharmacogenom* or pharmacogenet* or high-throughput nucleotide sequencing or immunochip*).ti,ab,kw.	274,881
#17	(gene* or genotyp* or phenotyp* or allele* or genom* or nucleotide) adj3 (polymorph* or variant* or mutat* or sequenc*).ti,ab,kw.	562,269
#18	or/7–17	1,944,116
#19	6 and 18	17,856
#20	exp allergy/	363,993
#21	exp anaphylaxis/	22,071
#22	exp hypersensitivity/	363,993
#23	exp skin rash/	8485
#24	exp adverse drug reaction/	124,786
#25	exp angio$edema/	6463
#26	exp Stevens-Johnson syndrome/	5702
#27	exp toxic epidermal necrolysis/	5702
#28	exp drug allergy/	48,203
#29	(immediat* or delay* immunoglobulin or immun* or IgE or Gell-Coombs or skin or cutaneous) adj4 (hypersensitivity or reaction* or rash).ti,ab,kw.	79,692
#30	(allergy or anaphyla* angio$edema or bronchospasm or cardiovascular collapse or urticaria or adverse drug reaction* or ADR* or severe cutaneous adverse reaction or SCAR or acute generalized exanthematous pustulosis or AGEP or drug reaction with eosinophilia and systemic symptoms or DRESS or Stevens-Johnson syndrome or SJS or toxic epidermal necrolysis or TEN or SJS-TEN or lichenoid drug eruption or maculopapular exanthema or benign exanthema or h$emolytic a$nemia or thrombocytopenia or petechia or serum sickness or vessel vasculitis or arthus reaction*).ti,ab,kw.	411,685
#31	or/20–30	901,659
#32	19 and 31	571
#33	(news or newspaper article or comment or editorial or interview or letter or review or systematic review or case report or case series).pt.	5,241,969
#34	32 not 33	498
#35	limit 34 to human	346

**Table A2:** Pre-specified search strategy for EMBASE via Elsevier.

Search	Query	Items Found
#1	penicillin*/exp AND [embase]/lim	140,066
#2	beta-lactam*/exp AND [embase]/lim	67,837
#3	cephalosporin*/exp AND [embase]/lim	63,568
#4	(penicillin*:ti,ab,kw,rn OR aminopenicillin*:ti,ab,kw,rn OR thiazolidine*:ti,ab,kw,rn OR ‘beta-lactam*’:ti,ab,kw,rn OR ‘beta lactam*’:ti,ab,kw,rn OR betalactam*:ti,ab,kw,rn OR lactam*:ti,ab,kw,rn OR cephalosporin*:ti,ab,kw,rn OR aminocephalosporin*:ti,ab,kw,rn OR carbapenem*:ti,ab,kw,rn OR aztreonam*:ti,ab,kw,rn OR monobactam*:ti,ab,kw,rn) AND [embase]/lim	141,126
#5	#1 OR #2 OR #3 OR #4	250,468
#6	gene*/exp AND [embase]/lim	8,237,173
#7	genotype*/exp AND [embase]/lim	472,522
#8	phenotyp*/exp AND [embase]/lim	862,932
#9	allele*/exp AND [embase]/lim	308,359
#10	genom*/exp AND [embase]/lim	1,017,291
#11	‘genome-wide association stud*’/exp AND [embase]/lim	52,473
#12	‘single nucleotide polymorphism*’/exp AND [embase]/lim	194,972
#13	‘human leukocyte antigen’/exp AND [embase]/lim	9
#14	Pharmacogenetic*/exp AND [embase]/lim	33,697
#15	(‘genome-wide association stud*’:ti,ab,kw OR GWAS:ti,ab,kw OR ‘single nucleotide polymorphism*’:ti,ab,kw OR SNP:ti,ab,kw OR ‘human leukocyte antigen’:ti,ab,kw OR HLA:ti,ab,kw OR haplotype*:ti,ab,kw OR pharmacogenom*:ti,ab,kw OR pharmacogenet*:ti,ab,kw OR ‘high-throughput nucleotide sequenc*’:ti,ab,kw OR immunochip*:ti,ab,kw) AND [embase]/lim	363,964
#16	((gene*:ti,ab,kw OR genotyp*:ti,ab,kw OR phenotyp*:ti,ab,kw OR allele*:ti,ab,kw OR genom*:ti,ab,kw OR nucleotide:ti,ab,kw) AND (polymorph*:ti,ab,kw OR variant*:ti,ab,kw OR mutat*:ti,ab,kw OR sequenc*:ti,ab,kw)) AND [embase]/lim	1,647,298
#17	#6 OR #7 OR #8 OR #9 OR #10 OR #11 OR #12 OR #13 OR #14 OR #15 OR #16	8,757,144
#18	#5 AND #16	20,352
#19	allergy/exp AND [embase]/lim	74,127
#20	anaphyla*/exp AND [embase]/lim	64,253
#21	hypersensitivity/exp AND [embase]/lim	600,339
#22	‘skin rash’/exp AND [embase]/lim	125,231
#23	‘adverse drug reaction*’/exp AND [embase]/lim	1,208,505
#24	angio?edema/exp AND [embase]/lim	670
#25	‘Stevens-Johnson syndrome’/exp AND [embase]/lim	10,675
#26	‘toxic epidermal necrolysis’/exp AND [embase]/lim	8494
#27	‘drug allergy’/exp AND [embase]/lim	56,025
#28	((immediat*:ti,ab,kw OR delay*:ti,ab,kw OR immunoglobulin:ti,ab,kw OR immun*:ti,ab,kw OR IgE:ti,ab,kw OR ‘Gell-Coombs’:ti,ab,kw OR skin:ti,ab,kw OR cutaneous) AND (hypersensitivity:ti,ab,kw OR reaction*:ti,ab,kw OR rash)) AND [embase]/lim	391,702
#29	(allergy:ti,ab,kw OR anaphyla*:ti,ab,kw OR angio?edema:ti,ab,kw OR bronchospasm:ti,ab,kw OR ‘cardiovascular collapse’:ti,ab,kw OR urticaria:ti,ab,kw OR ‘adverse drug reaction*’:ti,ab,kw OR ADR*:ti,ab,kw OR ‘severe cutaneous adverse reaction*’:ti,ab,kw OR SCAR:ti,ab,kw OR ‘acute generalized exanthematous pustulosis’:ti,ab,kw OR AGEP:ti,ab,kw OR ‘drug reaction with eosinophilia and systemic symptom*’:ti,ab,kw OR DRESS:ti,ab,kw OR ‘Stevens-Johnson syndrome’:ti,ab,kw OR SJS:ti,ab,kw OR ‘toxic epidermal necrolysis’:ti,ab,kw OR TEN:ti,ab,kw OR ‘SJS-TEN’:ti,ab,kw OR ‘lichenoid drug eruption’:ti,ab,kw OR ‘maculopapular exanthema’:ti,ab,kw OR ‘benign exanthema’:ti,ab,kw OR ‘h?emolytic a?nemia’:ti,ab,kw OR thrombocytopenia:ti,ab,kw OR petechia:ti,ab,kw OR ‘serum sickness’:ti,ab,kw OR ‘vessel vasculitis’:ti,ab,kw OR ‘arthus reaction*’:ti,ab,kw) AND [embase]/lim	1,023,837
#30	#19 OR #20 OR #21 OR #22 OR #23 OR #24 OR #25 OR #26 OR #27 OR #28 OR #29	2,743,793
#31	#18 AND #30	1146
#32	(news:it OR ‘newspaper article’:it OR comment:it OR editorial:it OR interview:it OR letter:it OR review:it OR ‘systematic review’:it OR ‘case report’:it OR ‘case series’:it) AND [embase]/lim	3,601,286
#33	#31 NOT #32	951

**Table A3:** Pre-specified search strategy for PubMED.

Search	Query	Items Found
#1	beta-lactam*[Pharmacological Action]	10,775
#2	((penicillin[MeSH Terms]) OR (beta-lactam[MeSH Terms])) OR (beta lactamase, cephalosporin[MeSH Terms])	134,109
#3	penicillin*[Title/Abstract] OR aminopenicillin*[Title/Abstract] OR thiazolidine*[Title/Abstract] OR “beta-lactam*”[Title/Abstract] OR betalactam*[Title/Abstract] OR lactam*[Title/Abstract] OR cephalosporin*[Title/Abstract] OR aminocephalosporin*[Title/Abstract] OR carbapenem*[Title/Abstract] OR aztreonam*[Title/Abstract] OR monobactam*[Title/Abstract]	138,945
#4	#1 OR #2 OR #3	206,937
#5	(((((((associations, genotype phenotype[MeSH Terms]) OR (allele[MeSH Terms])) OR (association study, genome wide[MeSH Terms])) OR (single nucleotide polymorphism[MeSH Terms])) OR (antigens, human leukocyte[MeSH Terms])) OR (analyses, genetic linkage[MeSH Terms])) OR (pharmacogenetics[MeSH Terms])) OR (pharmacogenomics[MeSH Terms])	365,641
#6	“genome-wide association stud*”[Title/Abstract] OR GWAS[Title/Abstract] OR “single nucleotide polymorphism*”[Title/Abstract] OR SNP[Title/Abstract] OR “human leukocyte antigen”[Title/Abstract] OR HLA[Title/Abstract] OR haplotype*[Title/Abstract] OR pharmacogenom*[Title/Abstract] OR pharmacogenet*[Title/Abstract] OR “high-throughput nucleotide sequenc*”[Title/Abstract] OR immunochip*[Title/Abstract]	295,433
#7	(gene*[Title/Abstract] OR genotyp*[Title/Abstract] OR phenotyp*[Title/Abstract] OR allele*[Title/Abstract] OR genom*[Title/Abstract] OR nucleotide[Title/Abstract]) AND (polymorph*[Title/Abstract] OR variant*[Title/Abstract] OR mutat*[Title/Abstract] OR sequenc*[Title/Abstract])	1,590,969
#8	#5 OR #6 OR #7	1,804,414
#9	#4 AND #8	16,303
#10	((((((((((allergy and immunology[MeSH Terms]) OR (allergy, drug[MeSH Terms])) OR (anaphylactic reaction[MeSH Terms])) OR (anaphylactic shock[MeSH Terms])) OR (anaphylaxis[MeSH Terms])) OR (skin rash[MeSH Terms])) OR (adverse drug reaction[MeSH Terms])) OR (angioedema[MeSH Terms])) OR (stevens johnson syndrome[MeSH Terms])) OR (toxic epidermal necrolysis[MeSH Terms])) OR (drug allergy[MeSH Terms])	172,981
#11	(immediat*[Title/Abstract] OR delay*[Title/Abstract] OR immunoglobulin[Title/Abstract] OR immun*[Title/Abstract] OR IgE[Title/Abstract] OR “Gell-Coombs”[Title/Abstract] OR skin[Title/Abstract] OR cutaneous[Title/Abstract]) AND (hypersensitivity[Title/Abstract] OR reaction*[Title/Abstract] OR rash[Title/Abstract])	320,731
#12	allergy[Title/Abstract] OR anaphyla*[Title/Abstract] OR angio?edema[Title/Abstract] OR bronchospasm[Title/Abstract] OR “cardiovascular collapse”[Title/Abstract] OR urticaria[Title/Abstract] OR “adverse drug reaction*”[Title/Abstract] OR ADR*[Title/Abstract] OR “severe cutaneous adverse reaction*”[Title/Abstract] OR SCAR[Title/Abstract] OR “acute generalized exanthematous pustulosis”[Title/Abstract] OR AGEP[Title/Abstract] OR “drug reaction with eosinophilia*”[Title/Abstract] OR DRESS[Title/Abstract] OR “Stevens-Johnson syndrome”[Title/Abstract] OR SJS[Title/Abstract] OR “toxic epidermal necrolysis”[Title/Abstract] OR TEN[Title/Abstract] OR “SJS-TEN”[Title/Abstract] OR “lichenoid drug eruption”[Title/Abstract] OR “maculopapular exanthema”[Title/Abstract] OR “hemolytic anemia”[Title/Abstract] OR thrombocytopenia[Title/Abstract] OR petechia[Title/Abstract] OR “serum sickness”[Title/Abstract] OR “vessel vasculitis”[Title/Abstract] OR “arthus reaction*”[Title/Abstract]	599,466
#13	#10 OR #11 OR #12	1,006,088
#14	#9 AND #13	630
#15	Filters applied: Humans.	429

**Table A4:** Pre-specified search strategy for Cochrane Library (CENTRAL).

Search	Query	Items Found
#1	MeSH descriptor: [Penicillins] explode all trees	5813
#2	MeSH descriptor: [beta-Lactams] explode all trees	9757
#3	MeSH descriptor: [Cephalosporins] explode all trees	4460
#4	(penicillin* OR aminopenicillin* OR thiazolidine* OR “beta-lactam*” OR betalactam* OR lactam* OR cephalosporin* OR aminocephalosporin* OR carbapenem* OR aztreonam* OR monobactam*):ti,ab,kw	9817
#5	#1 OR #2 OR #3 OR #4	15,324
#6	MeSH descriptor: [Genes] explode all trees	1710
#7	MeSH descriptor: [Genotype] explode all trees	4750
#8	MeSH descriptor: [Phenotype] explode all trees	1529
#9	MeSH descriptor: [Alleles] explode all trees	747
#10	MeSH descriptor: [Genome] in all MeSH products	2125
#11	MeSH descriptor: [Genome-Wide Association Study] explode all trees	177
#12	MeSH descriptor: [Polymorphism, Genetic] explode all trees	3159
#13	MeSH descriptor: [Polymorphism, Single Nucleotide] explode all trees	1412
#14	MeSH descriptor: [HLA Antigens] explode all trees	684
#15	MeSH descriptor: [Pharmacogenomic Testing] explode all trees	61
#16	(“genome-wide association stud*” OR GWAS OR “single nucleotide polymorphism*” OR SNP OR “human leukocyte antigen” OR HLA OR haplotype* OR pharmacogenom* OR pharmacogenet* OR “high-throughput nucleotide sequenc*” OR immunochip*):ti,ab,kw	10,780
#17	(gene* OR genotyp* OR phenotyp* OR allele* OR genom* OR nucleotide):ti,ab,kw AND (polymorph* OR variant* OR mutat* OR sequenc*):ti,ab,kw	31,649
#18	#6 OR #7 OR #8 OR #9 OR #10 OR #11 OR #12 OR #13 OR #14 OR #15 OR #16 OR #17	40,804
#19	#5 AND #18	340
#20	MeSH descriptor: [Hypersensitivity] explode all trees	21,652
#21	MeSH descriptor: [Drug Hypersensitivity] explode all trees	1004
#22	MeSH descriptor: [Anaphylaxis] explode all trees	195
#23	MeSH descriptor: [Exanthema] explode all trees	219
#24	MeSH descriptor: [Drug-Related Side Effects and Adverse Reactions] explode all trees	3784
#25	MeSH descriptor: [Angioedema] explode all trees	235
#26	MeSH descriptor: [Stevens-Johnson Syndrome] explode all trees	28
#27	(immediat* OR delay* OR immunoglobulin OR immun* OR IgE OR “Gell-Coombs” OR skin OR cutaneous):ti,ab,kw AND (hypersensitivity OR reaction* OR rash):ti,ab,kw	42,726
#28	(allergy OR anaphyla* OR angio?edema OR bronchospasm OR “cardiovascular collapse” OR urticaria OR “adverse drug reaction*” OR ADR* OR “severe cutaneous adverse reaction*” OR SCAR OR “acute generalized exanthematous pustulosis” OR AGEP OR “drug reaction with eosinophilia and systemic symptom*” OR DRESS OR “Stevens-Johnson syndrome” OR SJS OR “toxic epidermal necrolysis” OR TEN OR “SJS-TEN” OR “lichenoid drug eruption” OR “maculopapular exanthema” OR “benign exanthema” OR “h?emolytic a?nemia” OR thrombocytopenia OR petechia OR “serum sickness” OR “vessel vasculitis” OR “arthus reaction*”):ti,ab,kw	150,078
#29	#20 OR #21 OR #22 OR #23 OR #24 OR #25 OR #26 OR #27 OR #28	186,410
#30	#19 AND #29	56
#31	#19 AND #29 in Trials	52

**Table A5:** Pre-specified search strategy for Web of Science.

Search	Query	Items Found
#1	TS = (penicillin* OR aminopenicillin* OR thiazolidine* OR “beta-lactam*” OR betalactam* OR lactam* OR cephalosporin* OR aminocephalosporin* OR carbapenem* OR aztreonam* OR monobactam*)	101,426
#2	TS = (gene* OR genotyp* OR phenotyp* OR allele* OR “genome-wide association stud*” OR GWAS OR “single nucleotide polymorphism*” OR SNP OR “human leukocyte antigen” OR HLA OR haplotype* OR pharmacogenom* OR pharmacogenet* OR “high-throughput nucleotide sequenc*” OR immunochip*)	8,347,106
#3	TS = ((gene* OR genotyp* OR phenotyp* OR allele* OR genom* OR nucleotide) AND (polymorph* OR variant* OR mutat* OR sequenc*))	1,694,996
#4	#2 OR #3	8,419,603
#5	#1 AND #4	42,017
#6	TS = ((immediat* OR delay* OR immunoglobulin OR immun* OR IgE OR “Gell-Coombs” OR skin OR cutaneous) AND (hypersensitivity OR reaction* OR rash))	243,436
#7	TS = (hypersensitivity OR allergy OR anaphyla* OR angio?edema OR bronchospasm OR “cardiovascular collapse” OR urticaria OR “adverse drug reaction*” OR ADR* OR “severe cutaneous adverse reaction*” OR SCAR OR “acute generalized exanthematous pustulosis” OR AGEP OR “drug reaction with eosinophilia and systemic symptom*” OR DRESS OR “Stevens-Johnson syndrome” OR SJS OR “toxic epidermal necrolysis” OR TEN OR “SJS-TEN” OR “lichenoid drug eruption” OR exanthema OR “maculopapular exanthema” OR “benign exanthema” OR “h?emolytic a?nemia” OR thrombocytopenia OR petechia OR “serum sickness” OR “vessel vasculitis” OR “arthus reaction*”)	788,981
#8	#6 OR #7	982,116
#9	#5 AND #8	2064
#10	TS = (animal OR in vivo OR in vitro)	2,660,720
#11	#9 NOT #10	1623
#12	#9 NOT #10 and Review Articles or Letters or Book Chapters or Editorial Materials (Exclude − Document Types)	1401

**Table A6:** Pre-specified search strategy for Scopus.

Search	Query	Items Found
#1	TITLE-ABS-KEY (penicillin* OR aminopenicillin* OR thiazolidine* OR “beta-lactam*” OR betalactam* OR lactam* OR cephalosporin* OR aminocephalosporin* OR carbapenem* OR aztreonam* OR monobactam*)	346,431
#2	TITLE-ABS-KEY (gene* OR genotyp* OR phenotyp* OR allele* OR “genome-wide association stud*” OR GWAS OR “single nucleotide polymorphism*” OR SNP OR “human leukocyte antigen” OR HLA OR haplotype* OR pharmacogenom* OR pharmacogenet* OR “high-throughput nucleotide sequenc*” OR immunochip*)	16,100,492
#3	TITLE-ABS-KEY ((gene* OR genotyp* OR phenotyp* OR allele* OR genom* OR nucleotide) AND (polymorph* OR variant* OR mutat* OR sequenc*))	3,230,900
#4	#2 OR #3	16,214,139
#5	#1 AND #4	98,514
#6	TITLE-ABS-KEY ((immediat* OR delay* OR immunoglobulin OR immun* OR IgE OR “Gell-Coombs” OR skin OR cutaneous) AND (hypersensitivity OR reaction* OR rash))	1,110,245
#7	TITLE-ABS-KEY (hypersensitivity OR allergy OR anaphyla* OR angio?edema OR bronchospasm OR “cardiovascular collapse” OR urticaria OR “adverse drug reaction*” OR ADR* OR “severe cutaneous adverse reaction*” OR SCAR OR “acute generalized exanthematous pustulosis” OR AGEP OR “drug reaction with eosinophilia and systemic symptom*” OR DRESS OR “Stevens-Johnson syndrome” OR SJS OR “toxic epidermal necrolysis” OR TEN OR “SJS-TEN” OR “lichenoid drug eruption” OR exanthema OR “maculopapular exanthema” OR “benign exanthema” OR “h?emolytic a?nemia” OR thrombocytopenia OR petechia OR “serum sickness” OR “vessel vasculitis” OR “arthus reaction*”)	2,291,033
#8	#6 OR #7	3,142,375
#9	#5 AND #8	11,518
#10	TITLE-ABS-KEY (animal OR in vivo OR in vitro)	2,391,537
#11	#9 AND NOT #10	10,091
#12	#11 AND NOT (TITLE-ABS-KEY (animal OR in AND vivo OR in AND vitro)) AND (EXCLUDE (DOCTYPE, “re”) OR EXCLUDE (DOCTYPE, “le”) OR EXCLUDE (DOCTYPE, “no”) OR EXCLUDE (DOCTYPE, “sh”) OR EXCLUDE (DOCTYPE, “ed”) OR EXCLUDE (DOCTYPE, “ch”) OR EXCLUDE (DOCTYPE, “tb”) OR EXCLUDE (DOCTYPE, “er”))	7741

**Table A7:** Pre-specified search strategy for CINAHL.

Search	Query	Items Found
#1	MJ (penicillin OR beta lactam antibiotics OR cephalosporins)	1148
#2	AB (penicillin* OR aminopenicillin* OR thiazolidine* OR “beta-lactam*” OR betalactam* OR lactam* OR cephalosporin* OR aminocephalosporin* OR carbapenem* OR aztreonam* OR monobactam*)	10,695
#3	S1 OR S2	11,385
#4	MJ (genetic OR genes OR genotype OR phenotype OR allele OR genome OR genome-wide association studies OR GWAS OR polymorphism, genetic OR single nucleotide polymorphism OR SNP OR human leucocyte antigen OR pharmacogenomics OR pharmacogenetics)	79,925
#5	AB (“genome-wide association stud*” OR GWAS OR “single nucleotide polymorphism*” OR SNP OR “human leukocyte antigen” OR HLA OR haplotype* OR pharmacogenom* OR pharmacogenet* OR “high-throughput nucleotide sequenc*” OR immunochip*)	23,753
#6	AB ((gene* OR genotyp* OR phenotyp* OR allele* OR genom* OR nucleotide) AND (polymorph* OR variant* OR mutat* OR sequenc*))	92,710
#7	S4 OR S5 OR S6	152,179
#8	S3 AND S7	1083
#9	MJ (allergy or allergies OR hypersensitivity reactions OR anaphylaxis OR drug allergy OR skin rash OR adverse drug reactions OR angioedema OR stevens-johnson syndrome OR toxic epidermal necrolysis)	5895
#10	AB ((immediat* OR delay* OR immunoglobulin OR immun* OR IgE OR “Gell-Coombs” OR skin OR cutaneous) AND (hypersensitivity OR reaction* OR rash))	28,792
#11	AB (allergy OR anaphyla* OR angio?edema OR bronchospasm OR “cardiovascular collapse” OR urticaria OR “adverse drug reaction*” OR ADR* OR “severe cutaneous adverse reaction*” OR SCAR OR “acute generalized exanthematous pustulosis” OR AGEP OR “drug reaction with eosinophilia and systemic symptom*” OR DRESS OR “Stevens-Johnson syndrome” OR SJS OR “toxic epidermal necrolysis” OR TEN OR “SJS-TEN” OR “lichenoid drug eruption” OR “maculopapular exanthema” OR “benign exanthema” OR “h?emolytic a?nemia” OR thrombocytopenia OR petechia OR “serum sickness” OR “vessel vasculitis” OR “arthus reaction*”)	373,229
#12	S9 OR S10 OR S11	398,516
#13	S8 AND S12	144
#14	MW (animal OR in vivo OR in vitro)	178,867
#15	S13 NOT S14	127
#16	Expanders: Apply equivalent subjects Source Types: Academic Journals	126
